# The effect of two weeks of spinal manipulative therapy and home stretching exercises on pain and disability in patients with persistent or recurrent neck pain; a randomized controlled trial

**DOI:** 10.1186/s12891-021-04772-x

**Published:** 2021-10-27

**Authors:** Anders Galaasen Bakken, Andreas Eklund, Anna Warnqvist, Søren O’Neill, Iben Axén

**Affiliations:** 1 Department of Environmental Medicine, Division of Intervention and Implementation Research for Worker Health, Karolinska Institutet, Nobels väg 13, S-, 171 77 Stockholm, Sweden; 2 Division of Biostatistics, Karolinska Institutet, Nobels väg 13, S-, 171 77 Stockholm, Sweden; 3 Spine Centre Southern Denmark, University Hospital of Southern Denmark, Østre Hougvej 55, 5500 Middelfart, Denmark

**Keywords:** Manipulative therapy, Stretching exercises, Pain, Disability, RCT, NRS-11, EQ5D, Neck disability index, McGill pain questionnaire

## Abstract

**Background:**

Recurrent or persistent neck pain affects a vast number of people globally, leading to reduced quality of life and high societal costs. Clinically, it is a difficult condition to manage, and treatment effect sizes are often moderate at best. Activity and manual therapy are first-line treatment options in current guidelines. We aimed to investigate the combination of home stretching exercises and spinal manipulative therapy in a multicentre randomized controlled clinical trial, carried out in multidiscipline ary primary care clinics.

**Methods:**

The treatment modalities utilized were spinal manipulative therapy and home stretching exercises compared to home stretching exercises alone. Both groups received 4 treatments for 2 weeks. The primary outcome was pain, where the subjective pain experience was investigated by assessing pain intensity (NRS − 11) and the quality of pain (McGill Pain Questionnaire). Neck disability and health status were secondary outcomes, measured using the Neck Disability Indexthe EQ-5D, respectively. One hundred thirty-one adult subjects were randomized to one of the two treatment groups. All subjects had experienced persistent or recurrent neck pain the previous 6 months and were blinded to the other group intervention. The clinicians provided treatment for subjects in both group and could not be blinded. The researchers collecting data were blinded to treatment allocation, as was the statistician performing data analyses. An intention-to-treat analysis was used.

**Results:**

Sixty-six subjects were randomized to the intervention group, and sixty-five to the control group. For NRS − 11, a B-coefficient of − 0,01 was seen, indication a 0,01 improvement for the intervention group in relation to the control group at each time point with a *p*-value of 0,305. There were no statistically significant differences between groups for any of the outcome measures.

**Conclusion:**

Based on the current findings, there is no additional treatment effect from adding spinal manipulative therapy to neck stretching exercises over 2 weeks for patients with persistent or recurrent neck pain.

**Trial registration:**

The trial was registered 03/07/2018 at ClinicalTrials.gov, registration number: NCT03576846.

**Supplementary Information:**

The online version contains supplementary material available at 10.1186/s12891-021-04772-x.

## Background

Musculoskeletal pain is responsible for the third-largest number of years lived with disability worldwide [1]. Its prevalence will likely rise due to an expected increase of such conditions in low-income and middle-income countries in the coming years [2] and a longer average life span worldwide [3].

Persistent or recurrent (also described as “chronic”) pain is a prevalent condition, affecting 20% of the population globally [4, 5]. Neck pain (NP) is a significant contributor to this group [6], as the condition is persistent or recurrent in 19–37% of the cases [7, 8]. The consequences for NP sufferers are, among others, a higher risk of sick leave [9], a reduced ability to manage everyday life [10], and reduced mental and physical health-related quality of life [11].

NP is often labeled *non-specific* when no direct underlying cause is found, such as myelopathy or malignancy [12]. The pain is commonly thought to arise from pain-producing structures such as the myofascia, cervical facet joints, or the disc [12]. The cause of persistent NP, on the other hand, is not all that clear. Persistent NP now falls under the category of chronic primary pain [4], defined in the ICD-11 as “pain … that persists or recurs for longer than three months and is associated with significant emotional distress or significant functional disability (interference with activities of daily life and participation in social roles) and that cannot be better explained by another chronic pain condition” [4]. The consensus definition is that persistent or recurrent NP is located in the area of the neck, is constant or recurrent, with a minimum duration of 3 months [13–15].

There is little doubt that physical activity is beneficial for most persistent or recurrent pain patients [16, 17]. It is also shown that some passive treatments reduce pain and have a place in the management of this patient group [18, 19]. Recent guidelines for the management of persistent NP suggest multi-modal care such as stress self-management, manipulation, mobilization, soft tissue therapy, high-dose massage, supervised group exercise, supervised yoga, supervised strengthening exercises, or home exercises [20–23].

Considering the worldwide suffering and cost of musculoskeletal pain, investigating and developing management approaches for this patient group is essential. As multi-modal treatment strategies are recommended for patients with persistent NP, investigating the commonly used treatment modalities and combinations of these could be useful contributions in managing this global epidemic.

In a clinical setting, patients will usually be offered a combination of treatments, advice, and exercise [24]. However, the specific combination of home stretching exercises and spinal manipulative therapy (SMT) has not been investigated for recurrent or persistent NP. We hypothesize that combining these two interventions will give a better outcome on pain and disability than stretching alone.

## Method

### Setting and design

Part of the study aimed to examine Heart Rate Variability (HRV) and Conditioned Pain Modulation, but this will be reported in separate publications. This study aims to determine the effects of a two-week treatment series consisting of i) home stretching exercises and SMT versus ii) home stretching exercises alone on pain and disability in a population of patients with persistent or recurrent NP. A sample size of 120 subjects was chosen based on a power calculation of the primary outcome of HRV [25], to be published seperately. We expected few drop-outs and therefore aimed for 130.

The study was a multicenter randomized clinical trial, investigating adults with recurrent or persistent NP. Five multidisciplinary primary care rehab clinics in the Stockholm area recruited patients and contributed to the data collection. Recruitment began in October 2018 with the data collection ending in April 2020. The final follow-up questionnaires were answered in June 2020.

These clinics had chiropractors among their staff and were all part of the regional health service. This means that the patients paid part of the incurred fee (up to a maximum of 200 SEK/25 USD per visit) to see any health care professional. The patient fee was capped at a total of 1150 SEK, after which all further costs were covered by the Swedish health care system. This also applied to the subjects in this study. The chiropractors providing care in this study were all licensed by the Swedish National Board of Health and Welfare.

### Recruitment

Patients were recruited through one of several avenues:

i) Patients contacted the clinics directly and was informed about the ongoing study, ii) patients were referred from local general medical practices that had been informed about the ongoing study, iii) patients responded to project-specific online posts on the participating clinics’ Facebook pages, iiii) patients responded to the monthly news letter from the clinic including information on the study and iiiii) patients responded to project-specific in-print advertising in local newspapers.

The possible subjects were screened for eligibility over the phone by the primary researcher using a protocol from Hallman et al. [26].

### Inclusion criteria

- Presence of recurrent (at least one previous episode) and persistent (duration more than 6 months) NP [26]
- No chiropractic treatment the previous 3 months [27]
- Minimum 18 years of age
- Able to read and write Swedish.

### Exclusion criteria

Conditions or medications that would affect the HRV measurements, such as

- cardiovascular disease
- hypertension
- diabetes
- pregnancy
- obesity (BMI > 30)
- steroids
- β-blockers
- antidepressants.

Also, subjects were excluded if they had

- serious, competing diagnoses, e.g. cancer, infection and serious trauma
- contra-indications to spinal manipulation, e.g. recent development of headache and/or dizziness, previous drop-attacks or acute cervical radiculopathy.

### Inclusion procedure

Prior to inclusion, potential participants received verbal and written information concerning the study as per local ethics guidelines and were able to ask questions about the study to the principal researcher. Written, signed consent was obtained from all participants at this point.

Eligible subjects were booked in for all the study visits, 5 in total.

### Randomization

The randomization sequence, using a 1:1 allocation ratio in randomly permuted blocks of different sizes, was generated off-site by a statistician, using SPSS version 27 [28]. Consecutively numbered sealed opaque envelopes were then prepared by a research assistant.

### First and subsequent visits

A questionnaire was used to record demographic data and self-reported measurements (described below) at the baseline visit. The physical/biological baseline measurements were recorded by a research assistant before the patient saw his/her chiropractor. The chiropractor opened the allocation envelope and provided the allocated treatment modality.

All patients received four treatments in the study. In order to perform the follow-up measurements, participants were scheduled for five clinic visits and were measured again by the research assistant before their third and fifth treatment, respectively.

The procedures are extensively reported in the study protocol [25].

### Blinding

Upon inclusion, all subjects were told that two different but common treatment modalities with similar clinical effects were tested in the study, but they were blind to what treatment the other group was receiving. Participants underwent the same examinations and number of treatments and had the same opportunities for support and to ask questions concerning their pain – the only systematic difference between groups was the allocated treatment.

The clinicians could not be blinded as they provided treatment for subjects in both groups.

The researchers collecting data were blinded to treatment allocation, as was the statistician performing data analyses.

### Procedures

#### Interventions

All subjects were scheduled for four treatments over the course of 2 weeks. The intervention dose and period were chosen as previous research had shown this would be sufficient to see a change in pain levels in a similar patient group [29, 30].

Both groups received a program of home stretch exercises with a documented effect on persistent neck pain [31], supplied in a leaflet with pictures and instructions (Attachment 1.). All subjects were asked to report the stretching in an exercise diary to measure adherence to the intervention. The diary is also found as part of Attachment 1.

The intervention group received SMT in addition to home stretching exercises. SMT in this study was used as a term describing both mobilization and High Velocity Low Amplitude manipulation.

Mobilization and HVLA manipulation have similar effect sizes when used as a treatment for persistent NP in studies using pragmatic designs [32]. They are both favorable compared to other interventions [33], particularly in combination with multi-modal approaches [33, 34]. SMT combined with exercise has also been shown to improve the short term reduction of persistent NP, compared to exercise alone [18].

The treating chiropractor tailored treatment methods to suit each patient to adapt to factors like the subject’s age, other diseases, and pain location. The treatment were given to any part of the spine as decided by the treating clinician. After completing the study, the chiropractor recorded treatment techniques used in each case from their patients’ files.

### Outcome measures

#### Pain (primary outcome)

Pain intensity was quantified using an 11-point numeric rating scale (NRS-11), anchored by the descriptors ‘No pain’ (0) and ‘Worst pain imaginable’ [10], presented on paper and online questionnaires [35, 36]. The qualitative characteristics of pain was assessed using the McGill Pain Questionnaire [37, 38] and the composite EQ-5D questionnaire was used to assess secondary effects of pain, such as emotions, previous pain experiences, and the effect on daily life [39–41]. The questionnaires have been shown to be reliable and valid in Swedish [35–41].

#### Disability (secondary outcome)

Using the Neck Disability Index (NDI) [42], the perceived level of disability was quantified by a summary score of the pain’s effects on a patient’s daily life. The instrument consists of 10 questions with a maximum score of 50, where a higher score indicates higher degrees of perceived disability. The patient is asked to reflect on the degree of limitation in activities affected by their NP the previous week. The questionnaire is reliable and validated in Swedish [42].

#### Health-related quality of life (secondary outcome)

Health-related quality of life (HRQoL) reflects the overall persistent or recurrent pain experience. It was measured using EQ-5D, as it has been validated in patients living with persistent pain [43]. The EQ-5D defines the individual’s health status by a single summary index ranging from 0 to 1, where 0 corresponds to death, and 1 corresponds to full health [41, 44]. The questionnaire is reliable and validated in Swedish [40, 41].

### Adverse reactions

After the first visit, the subjects were asked to report any adverse reactions through a text message (SMS). They were asked whether they experienced a reaction to the first treatment, e.g. increased tenderness or fatigue in the neck, answered with an NRS-11 scale anchored by the descriptors ***‘***No reaction***’*** (0) and ***‘***Worst reaction imaginable***’*** [10] [45].

### Follow-up

Digital outcome questionnaires were administered on the day of the third (1 week after the first treatment) and fifth treatment (2–4 days after the fourth treatment), using a system managed by Survey & Report by Artologic [46] through Karolinska Institutet.

Repeated measures of pain intensity (NRS-11) were recorded daily during the 2 week study period using SMS-Track® [45].

### Ethics

This study investigated patients seeking care for their pain. A pure placebo trial was, therefore, not indicated [47]. As all subjects received home stretching exercises, a potentially beneficial intervention was provided. Four treatments were considered sufficient to detect a change, but not too burdensome if the patient did not benefit.

All study participants were assigned a unique number by a research assistant when included in the study. A code key connecting the participant’s ID and the unique number was stored according to the Swedish National Board of Health and Welfare’s requirements for the storage of journal documents.

All collected data will be stored for at least ten years, and individuals can only be identified with the code key. Only researchers directly affiliated with the project have access to the material.

The study was conducted in accordance with the Helsinki declaration [48]. Data was stored in accordance with European GDPR regulations.

### Statistical analysis

Continuous variables were reported with means and standard deviations, categorical variables were reported as counts and percentages.

Intention to treat analysis was used.

Several subjects reported the McGill Questionnaire to be challenging to complete due to the numerous alternatives for quality of pain. Due to time constraints at the baseline visit, the subjects were instructed to skip the questions that did not relate to their pain instead of discussing them with the research assistant. Therefore, if a question was not answered, the subject did not experience the particular pain sensation.

The NRS-11 data was incomplete with seven non-responses, of which four concurrent (same date) pain scores could be obtained from the McGill questionnaire. For the final 3 missing observations, the Last Observation Carried Forward (LOCF) was used. A method of multiple imputations with fully conditional specification and twenty imputation rounds was used for NDI and EQ-5D to produce unbiased estimates with appropriate coverage [49].

Repeated measurements using all available time points were analyzed using a linear mixed effects model with person specific random intercept. A quadratic model was also estimated but the quadratic term did not improve model fit, judged by AIC (Akaike Information Criteria) and BIC (Bayesian Information Criteria). The parameter of interest was the interaction between group allocation and time, which can be interpreted as the difference in speed of change between the groups. Two additional analyses were performed; one adjusted for baseline values, the other also included age and gender.

The graphical representations were done using a quadratic model to allow for more flexibility and easier visual interpretation of the time effect interaction.

The change over the 2 weeks was calculated for all four outcomes; pain intensity, effective quality of pain, disability and quality of life. A minimal clinically important difference (MCID) was based on previous studies [50–53] and calculated as the percentage of subjects in each group reaching MCID. The values were as following; NRS-11: 2 points or more, NDI: 10 points or more, McGill: More than 5 points, EQ-5D: 0,03 points or more. The difference between groups in the probability of attaining MCID was estimated using logistic regression.

For linear and logistic regression analysis, both an unadjusted model, and models adjusted for baseline differences, as well as for age and gender, were estimated. The reported side effects are reported as proportions. A per-protocol analysis was performed as a sensitivity analysis, following the same method as the primary analysis for repeated measures.

*P*-values smaller than .05 were considered significant. The analysis was performed using SPSS 27 [28] and Stata version 15 (StataCorp. 2017).

## Results

**Fig. 1:**
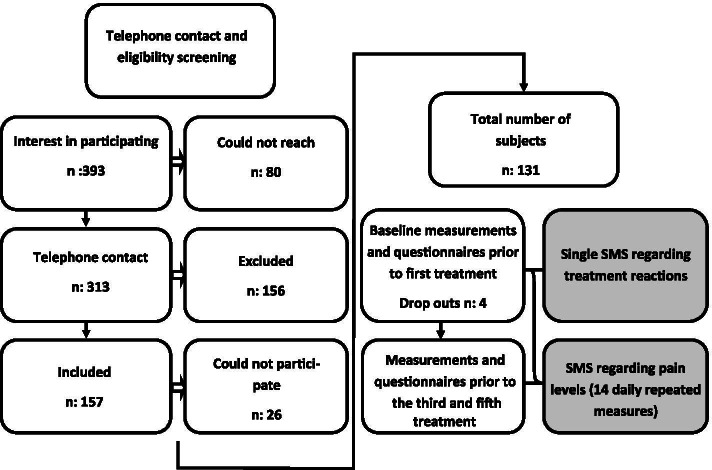
Flow chart of the recruitment process and obtained measurements

A total of 131 patients were included in the study. See attached flow chart (Fig. 1) for details.

Demographic and pain characteristics are presented in Table 1.

**Table 1:** Demographics and pain characteristics of the study population at baseline, *N* = 131

	*Intervention (66)*	*Control (65)*
*Age, Mean (sd)*	*57 (14,0)*	*57,5 (13,7)*
*Female, n (%)*	*37 (56)*	*36 (55)*
*Pain duration*		
*1. Less than 6 months, n (%)*	*0 (0)*	*1 (2)*
*2. 6–12 months, n (%)*	*8 (12)*	*10 (16)*
*3. Several years, n (%)*	*57 (88)*	*51 (82)*
*STarT Back categories*		
*1. Low risk, n (%)*	*47 (80)*	*48 (79)*
*2. Medium risk, n (%)*	*7 (12)*	*11 (18)*
*3 High risk, n (%)*	*5 (9)*	*2 (3)*
*If seeing a chiropractor before, how effective was it?*		
*1. Never seen a chiropractor before, n (%)*	*12 (19)*	*11 (17)*
*2. Good or excellent, n (%)*	*39 (60)*	*35 (55)*
*3. No difference, n (%)*	*14 (22)*	*17 (27)*
*4. Got worse, n (%)*	*0 (0)*	*1 (2)*
*Type of occupation*		
*1. No job, n (%)*	*19 (29)*	*20 (31)*
*2. Mostly hard labor, varied or standing, n (%)*	*17 (27)*	*15 (23)*
*3. Mostly sitting, n (%)*	*30 (46)*	*30 (46)*
*Similar pain previously*		
*1. No, n (%)*	*35 (53)*	*34 (56)*
*2. Yes, n (%)*	*31 (47)*	*27 (44)*
*Arm pain, n (%)*	*42 (65)*	*36 (57)*
*Pain in the midback, n (%)*	*39 (61)*	*37 (62)*
*Pain in the low back, n (%)*	*39 (62)*	*37 (59)*
*Sick leave the previous year*		
*Do not work, n (%)*	*13 (20)*	*18 (28)*
*No, n (%)*	*47 (71)*	*41 (63)*
*Yes, between 1 and 7 days, n (%)*	*3 (5)*	*2 (3)*
*Yes, between 8 and 14 days, n) (%)*	*3 (5)*	*0 (0)*
*Yes, more than 15 days, n (%)*	*0 (0)*	*4 (6)*
*Expect to improve (0–10), Mean (std)*	*6,0 (2,2)*	*5,8 (2,3)*

The two groups were similar at baseline, as reported in Table 1, except NRS-11, which differed with 0,51/10 points. An independent t-test showed this difference to be non-significant.

During the first week, four subjects dropped out, all in the control group. One dropped out due to a change in their work schedule following Covid-19, and two subjects were not pleased with receiving home stretching exercises only. One subject canceled for no reason. After the study period had ended, it was noted that two subjects had received treatment though they were part of the control group.

See Table 2 and supplementary files 1, 2, 3 and 4 for an overview of outcome measures at baseline and at two-week follow-up. Both groups showed improvements in pain intensity (NRS-11), affective quality of pain (McGill questionnaire), disability (NDI) and quality of life (EQ-5D).

**Table 2:** Mean effect and B-coefficient for all outcome measures

	Intervention	Control	B	CI		*P*-value
NRS-11						
Baseline, mean (n)	4,7 (65)	4,2 (64)				
After 2 weeks (128) (n)	3,6 (66)	3,1 (62)				
Difference BL-2 weeks	-1,1	-1,1	−0,0	−0,0	0,0	0,31
McGill questionnaire						
Baseline (n)	23,7 (64)	23,2 (64)				
After 2 weeks (n)	22,8 (65)	21,3 (61)				
Difference BL-2 weeks	−0,9	−1,9	0,5	−0,6	1,6	0,38
EQ5D						
Baseline (n)	0,897 (66)	0,903 (65)				
After 2 weeks (n)	0,897 (62)	0,899 (57)				
Difference BL-2 weeks	−0,007	0,006	0,001	− 0,012	0,013	0,92
Neck Disability Index						
Baseline (n)	22,6 (57)	21,7 (58)				
After 2 weeks (n)	20,5 (58)	19,8 (55)				
Difference BL − 2 weeks	-2,1	−1,9	−0,0	−0,3	0,1	0,45

There were no statistically significant differences between the two groups in change scores for any of the outcome measures.

Additional models were tested, first using baseline differences as a covariate, secondly age and gender. Finally, a per protocol analysis was conducted, neither changed the overall estimates or precision and are therefore not reported here.

In the Supplementary files 1, 2, 3 and 4, daily development of mean pain intensity (NRS-11), McGill scores, Neck Disability Index and EQ5D scores with Confidence Intervals for both groups from baseline to the end of the intervention period (2 weeks) are presented graphically. The graphs are produced using a quadratic model.

Besides the crude difference between follow-up and baseline, we also calculated the proportion of subjects that reached MCID for each outcome. As reported in Table 3, some subjects in both groups reached a MCID for all measurements ranging from 18 to 38%. There are no statistically significant differences between the two groups for any measurement. The adjusted analysis, including baseline, age and gender, is not included in the table as it did not change the overall effect and statistical significance.

**Table 3:** Proportion of individuals reaching minimal clinically important difference for the different outcome measures and the difference between groups

	Intervention	Control	OR	*P*-value
NRS-11				
Reached MCID, n (%)	24/64 (37)	22/59 (38)	1,0	0,98
McGill				
Reached MCID, n (%)	15/62 (24)	11/57 (18)	1,4	0,46
EQ5D				
Reached MCID, n (%)	14/66 (21)	19/65 (29)	0,6	0,30
NDI				
Reached MCID, n (%)	15/66 (22)	18/65 (28)	0,7	0,45

Further, a per-protocol-analysis did not significantly affect results (results not shown).

The adherence to the stretching protocol was good, as shown in Table 4. Training diaries from 90% of the entire study population were obtained. Of these, 77,8% in the intervention group and 76,4% in the control group adhered to the stretching regimen at least 13/14 days. All participants reported that they did their stretching exercises at least 10 out of 14 days.

**Table 4:** Adherence to stretching exercises as reported in exercise diaries

Days of doing stretching (out of 14)	10	11	12	13	14
Group					
Intervention, n (%)	4 (6,3)	0 (0,0)	10 (15,9)	10 (15,9)	39 (61,9)
Control, n (%)	0 (0,0)	7 (12,7)	6 (10,9)	6 (10,9)	36 (65,5)

### Adverse events

Four intense adverse events were reported in the study, defined by ≥8 (NRS-11) [54], three in the intervention group, and one in the control group. More adverse incidents were reported in the intervention group, with a mean pain intensity (NRS-11) of 2,75 compared to 1,22 in the control group. There were no statistically significant differences between the two groups.

## Discussion

The current study was undertaken to investigate whether four sessions of SMT combined with 2 weeks of stretching exercises was superior to stretching exercises alone, in a group of chronic NP patients. Both groups improved with treatment, but we found no statistical differences for any of our outcome measures between the two groups.

Previous studies have found a combination of home exercises and SMT superior to home exercises alone [19, 34], and most current guidelines recommend multi-modal care, including SMT and exercises [20–23]. However, the combined effect of SMT and home stretching exercises specifically has not been investigated in detail in chronic NP.

The stretching exercises used in this study were developed by Ylinen et al. [31] in a study comparing home stretching exercises with manual therapy. They found that both interventions considerably decreased neck pain and disability in women with persistent or recurrent neck pain. The difference in effectiveness between the interventions was minor. We hypothesized that SMT and home stretching exercises combined would have a better effect than home stretching exercises alone after a two-week treatment period. However, the results rather imply that providing stretching only is as effective as combining it with SMT, and that perhaps the patient’s own preference should guide the treatment choice.

A population with persistent or recurrent pain will typically have periodic changes in pain intensity [55], and the fluctuations in pain and disability in this study could be due to normal variations in pain. Overall, an improvement during the study for all measurements was seen in both groups when adjusted for baseline differences in pain intensity.

The number of people reaching MCID did not differ statistically between groups. Just below 40% reached this threshold in pain intensity (NRS-11), but only around 20% for the impact of pain quality (McGill questionnaire). This is an interesting observation as they are both measuring the subject’s pain experience. It appears that pain intensity is more readily affected by the intervention than the sensory and affective pain experience, measured using the McGill Questionnaire. However, an absolute consensus on MCID values does not exist, so care should be taken when comparing these values. It was not expected that a large part of the study population would reach MCID, considering the chronicity of their pain and the limitations of a two-week study period.

It was observed that the SMT and stretching group experienced more adverse reactions than the group receiving stretching only. The difference was not statistically significant, but is not surprising considering previous research. It is estimated that about 50% of patients experience minor to moderate adverse events after any manual treatment, particularlyafter the first visit [54]. Since the risk of major adverse events is low, and that these adverse events are known to be short-lasting [56], adverse events were only monitored after the first study visit. Adverse events in this study were few and mainly mild, in line with previous studies [54, 56] confirming that both interventions are safe.

Adherence to the home stretching exercises was good in both groups (all subjects reporting compliance at least 10 out of 14 days, and 77% 13 or 14 days). In the original stretching study, the investigator asked the subjects to perform the stretching exercises five times a week [31]. We chose to recommend the exercises to be performed daily as the intervention period was only 2 weeks. We also accounted for some forgetfulness among the subjects based on clinical experience. Previous research has shown that getting people to perform their rehab exercises is one of the most difficult challenges in clinical health interventions [57, 58]. We propose that adherence to the program was vital in the observed improvement. Filling in a diary probably improved adherence to the protocol.

For pain intensity (NRS-11), there was a difference between the treatment groups at baseline. This happened by chance in the randomization process and did not significantly affect the estimates when adjusted for.

### Methodological considerations

#### Weaknesses

It was impossible to blind the therapists performing the treatments. This could possibly lead to bias favouring the intervention group. However, the therapists received written and verbal instructions on how to interact in a similar fashion with both groups to minimize this risk. As there was no significant advantage of the intervention group, this potential bias was considered to be low.

Most of the participants had already been to the clinic at a previous time point. A possible selection bias may have occurred as many of the subjects already had a good experience with a previous chiropractic treatment. This is an unknown effect, not directly related to a specific treatment or clinician. If the subject had seen a therapist for their neck pain previously and experienced a good effect, this could have had a substantial impact on the expectations of a treatment effect. However, it can be argued that if a therapist was already managing a patient with neck pain successfully, i.e. they have a good relationship, and the patient is experiencing that the treatment regime helps them manage their life with pain, then participation in this study would not be needed. The improvement in pain and disability in the intervention and control groups was similar in this study. The first and obvious conclusion, is that the two treatments have equal clinical benefit to patients, which is not in line with previous publications [19, 34]. The current findings, however, could be affected by factors such as a flooring effect, the relatively short treatment period for such a chronic patient cohort, or the chronicity of this patient group.

We had no minimum inclusion value for baseline measurements, and generally speaking, the study population had low levels of pain and disability. This was not done to include all aspects of persistent or recurrent NP patients, as it is a diverse group. For NRS-11 i.e., a level of 1 or 2 out of 10 at baseline will have limited the improvement to 1 or 2 points, respectively. This could possibly have limited the difference between groups.

The inclusion criteria explicitly stated recurrent or persistent NP, where pain fluctuations are the norm [55]. As the recruiting was done up to 5 weeks before the intervention period, patients may have been in more pain when recruited but experienced a normal fluctuation for the better before the study started. Thus, changes seen in his study are probably not due to regression to mean, but this cannot be ruled out.

Four treatments were chosen as it has been seen from a previous study [29] that a definite improvement observed at the fourth treatment usually is related to an improvement after 3 months. However, that study population consisted of patients with low back pain, and among these, only a group had persistent or recurrent pain. Also, the subjects sought chiropractic treatment, with no limitations regarding treatment intervention. It is possible that persistent or recurrent NP is more resistant to SMT, and that our study population had lower expectations to the intervention as they did not actively seek care at a chiropractic clinic.

These weaknesses may explain why the observed effect in the study population differs from previous studies, exemplified by the study by Maiers et al. [34] that had a minimum NRS-11 of 3 as inclusion criteria on NRS-11 and tested an intervention lasting twelve weeks.

#### Strengths

Blinding seems to have been successful, and the research assistant and statistician were blinded to the group allocation. The study population was blinded to the other group intervention.

Both groups received the same amount of time and attention from the chiropractors. The control group underwent a standard palpation examination as if the chiropractor intended to give manual treatment. Advise and reassurance were given in both groups.

Measuring pain levels and the affective quality of pain is essential for capturing the chronic pain experience. Using multiple questionnaires, such as NRS-11, McGill questionnaire, and EQ-5D together covers the main aspects of chronic pain.

The response rate was excellent, with a small number of dropouts and an exercise diary to investigate compliance. This showed a strong dedication to the stretching regime.

### External validity

This was a pragmatic study, applicable for all clinicians working with persistent or recurrent NP patients. The study was perfomerd in a normal clinical setting, reflected in the heterogenic demographics among the study population. It mirrored standard treatment strategies to allow for conclusions on effect.

## Conclusion

Daily stretching exercises with and without added spinal manipulation were associated with some clinical improvement over a two-week period, but no significant difference in improvement was observed between groups.

## Supplementary Information

**Additional file 1.** Graph NRS11. Daily development of mean pain intensity (NRS-11) with Confidence Intervals for both groups from baseline to the end of the intervention period (2 weeks). The graph is presented using a quadratic model.

**Additional file 2..** Graph McGill. McGill scores with Confidence Intervals for both groups measured at baseline, 1 week, and 2 weeks. The graph is presented using a quadratic model.

**Additional file 3.** Graph NDI. Neck Disability Index scores with Confidence Intervals for both groups measured at baseline, 1 week and 2 weeks. The graph is presented using a quadratic model.

**Additional file 4.** Graph EQ5D. EQ5D scores with Confidence Intervals for both groups measured at baseline, 1 week, and 2 weeks. The graph is presented using a quadratic model.

**Additional file 5.** Stretch exercises to perform daily for 14 days.

## Data Availability

The data that support the findings of this study are available from Karolinska Institutet, but restrictions apply to the availability of these data, which were used under license for the current study, and so are not publicly available. Data are, however, available from the authors upon reasonable request and with permission of Karolinska Institutet.

## Abbreviations

NP: Neck Pain
SMT: Spinal Manipulative Therapy
HRV: Heart Rate Variability
NDI: Neck Disability Index
NRS-11: 11-point numeric rating scale
HRQoL: Health-related quality of life
LOCF: Last Observation Carried Forward
MCID: Minimal Important Clinical Difference
