# Supplementary material for: The effect of two weeks of spinal manipulative therapy and home stretching exercises on pain and disability in patients with persistent or recurrent neck pain; a randomized controlled trial
Source: BMC Musculoskelet Disord. 2021 Oct 27;22:903. doi: 10.1186/s12891-021-04772-x (PMC8549416; doi:10.1186/s12891-021-04772-x)

Figure 2. Daily development of mean pain intensity (NRS-11) with Confidence Intervals for both groups from baseline to the end of the intervention period (2 weeks). The graph is presented using a quadratic model.


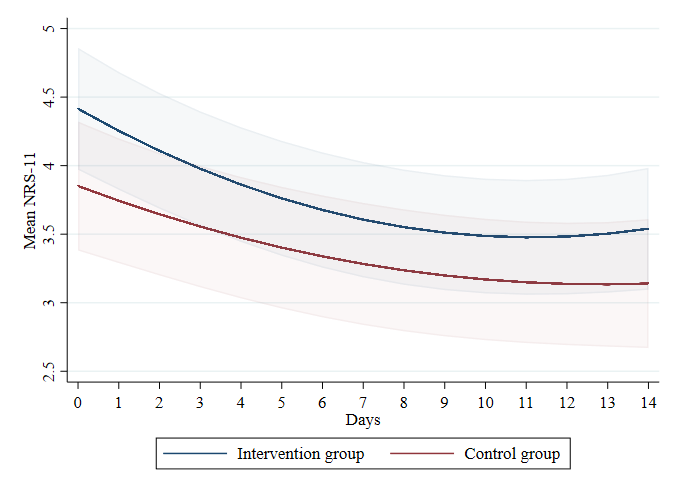

Supplement: Supplementary file 1 — Additional file 1. Graph NRS11. Daily development of mean pain intensity (NRS-11) with Confidence Intervals for both groups from baseline to the end of the intervention period (2 weeks). The graph is presented using a quadratic model. [file 12891_2021_4772_MOESM1_ESM.docx]
